# Supplementary material for: Pseudomonas aeruginosa PA14 produces R-bodies, extendable protein polymers with roles in host colonization and virulence
Source: Nat Commun. 2021 Jul 29;12:4613. doi: 10.1038/s41467-021-24796-0 (PMC8322103; doi:10.1038/s41467-021-24796-0)
Supplement: Supplementary file 1 — Supplementary Information [file 41467_2021_24796_MOESM1_ESM.pdf]

Suppl. Figure 1

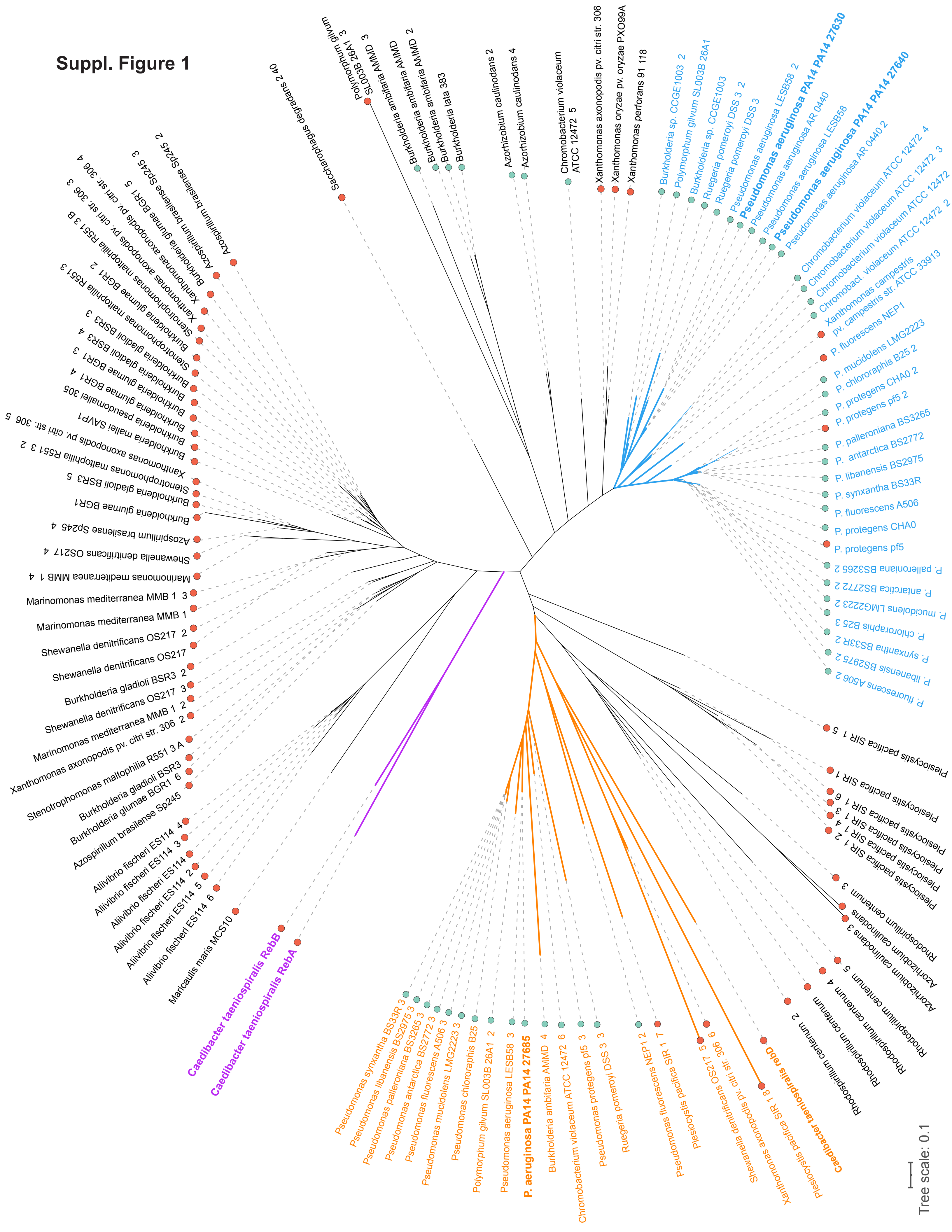

Supplementary Figure 1. Phylogenetic tree of proteins from 38 bacterial strains that are homologous to *C. taeniospiralis* RebB. The bacterial strains included are those with complete genomes from Figure 6 of Raymann *et al*. (1) plus 14 representative pseudomonads. *C. taeniospiralis* RebA and RebB are shown in purple. The cluster containing *C. taeniospiralis* RebD and its *Pseudomonas* homologs is shown in orange. We have assigned the designation “RebP” to Reb homologs that cluster with *P. aeruginosa* PA14_27630 and PA14_27640 (shown in blue). Green circles at the end of each branch indicate the presence of FecI2 homologs in the respective strains, while red circles indicate its absence.

Suppl. Figure 2

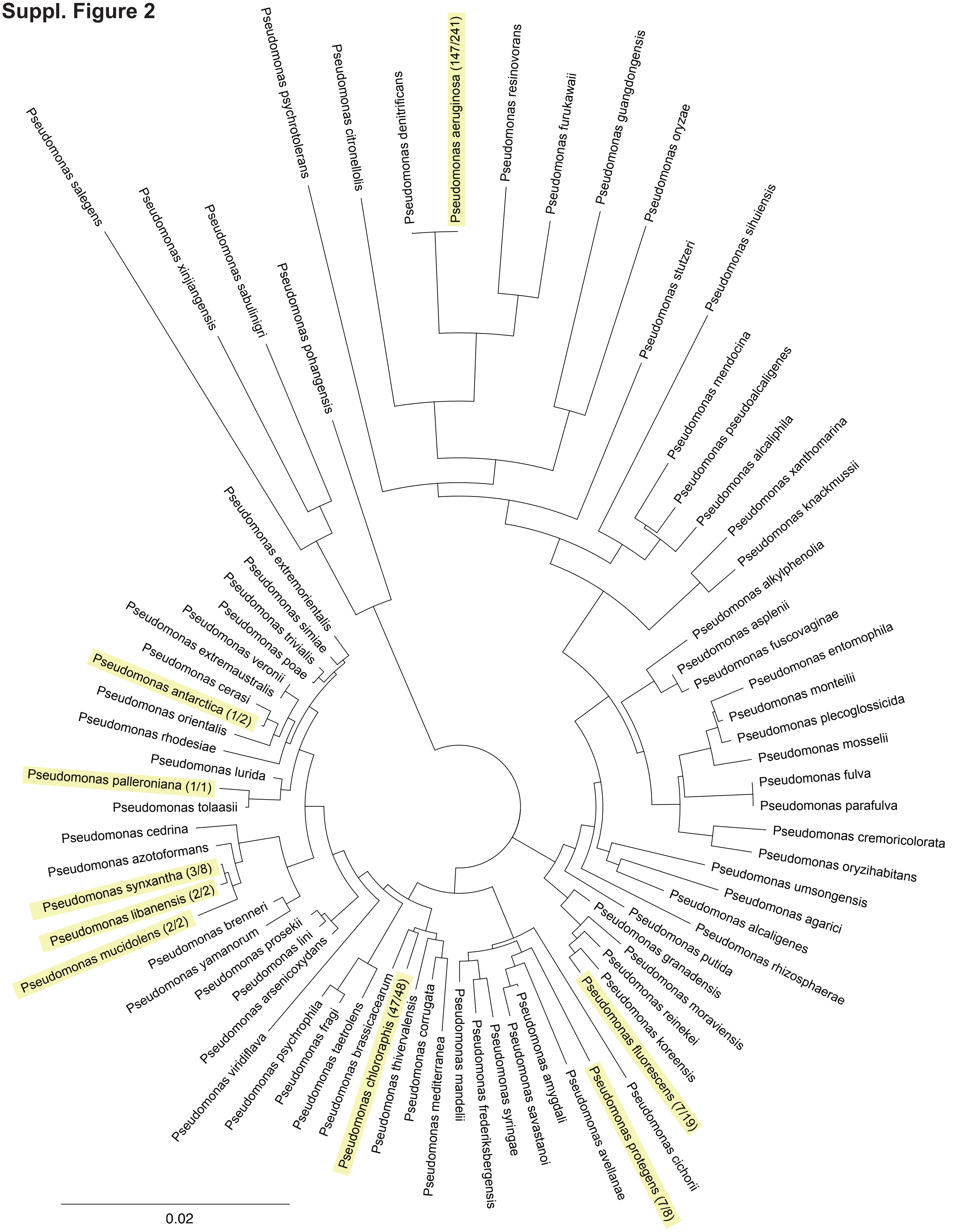

Supplementary Figure 2. 16S rRNA-based phylogenetic tree showing all *Pseudomonas* species with complete genomes available in the Pseudomonas Genome Database. Species with at least one strain containing one or more *rebP1* homolog(s) are highlighted in yellow. Indicated in parentheses are the number of strains with *rebP1* homologs out of the total number of strains with complete genomes for a respective species.

Suppl. Figure 3

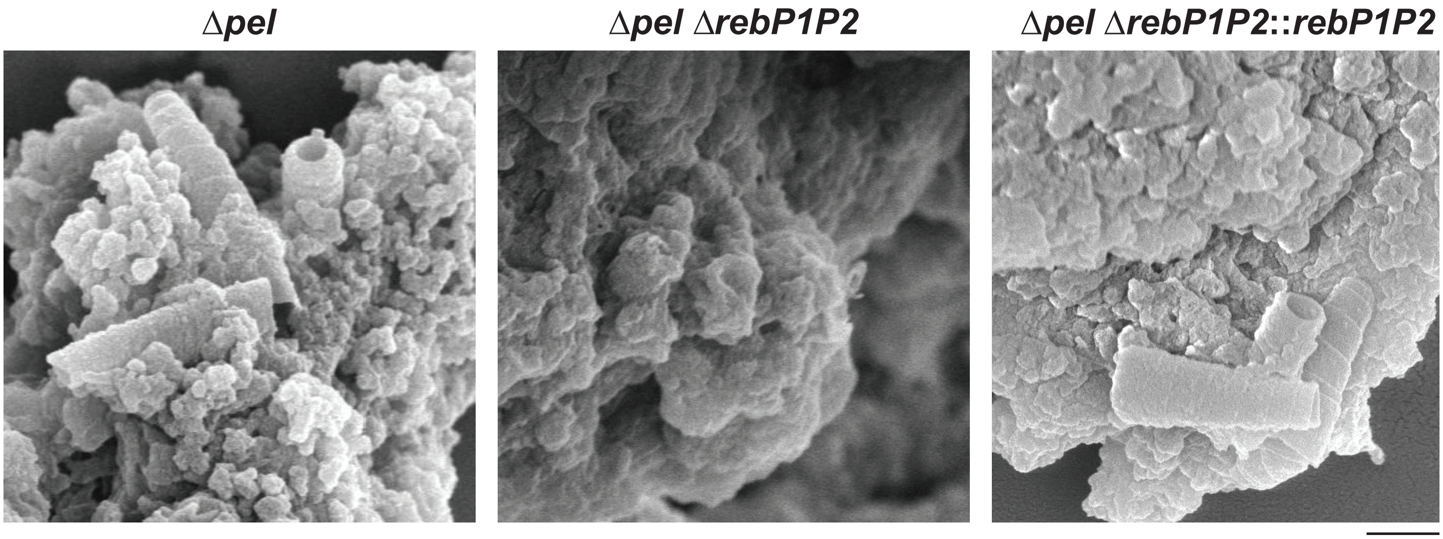

Supplementary Figure 3. SEM images of SDS-insoluble fractions prepared from biofilms of strains with the indicated mutations. The Δ*pel* background was used because this parent strain is more amenable to disruption. Scale bar is 500 nm. The images are representative of 16 fields of view captured for each strain from three experiments. For Δ*pel* and Δ*pel*Δ*rebP1P2*::*rebP1P2* samples, every field of view captured had an R-body present whereas none were detected in the Δ*pel*Δ*rebP1P2* sample. On average, 10.8 R-bodies were seen in each field of view (range from 1-40 R-bodies) for Δ*pel* and 9.6 R-bodies with a range of 1-30 R-bodies in Δ*pel*Δ*rebP1P2*::*rebP1P2*.

Suppl. Figure 4

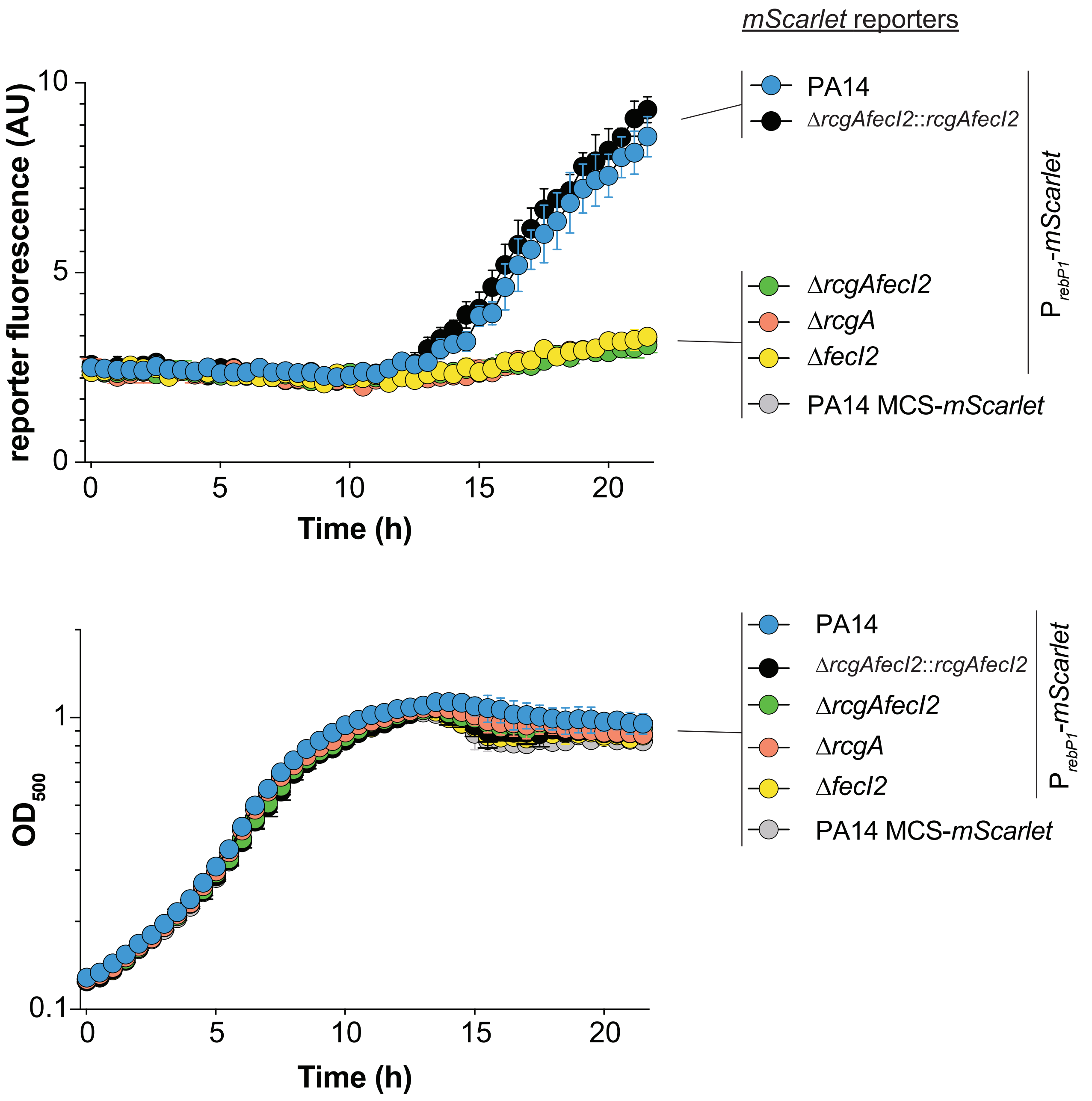

Supplementary Figure 4. Expression of mScarlet by the indicated genotypes containing the P_{rebP1} or promoterless transcriptional reporter in shaken 1% tryptone liquid cultures grown at 25 °C. Corresponding growth curves are shown in the bottom panel. n = 5 biological replicates from two experiments. Data are presented as mean values. Error bars represent standard deviation.

Suppl. Figure 5

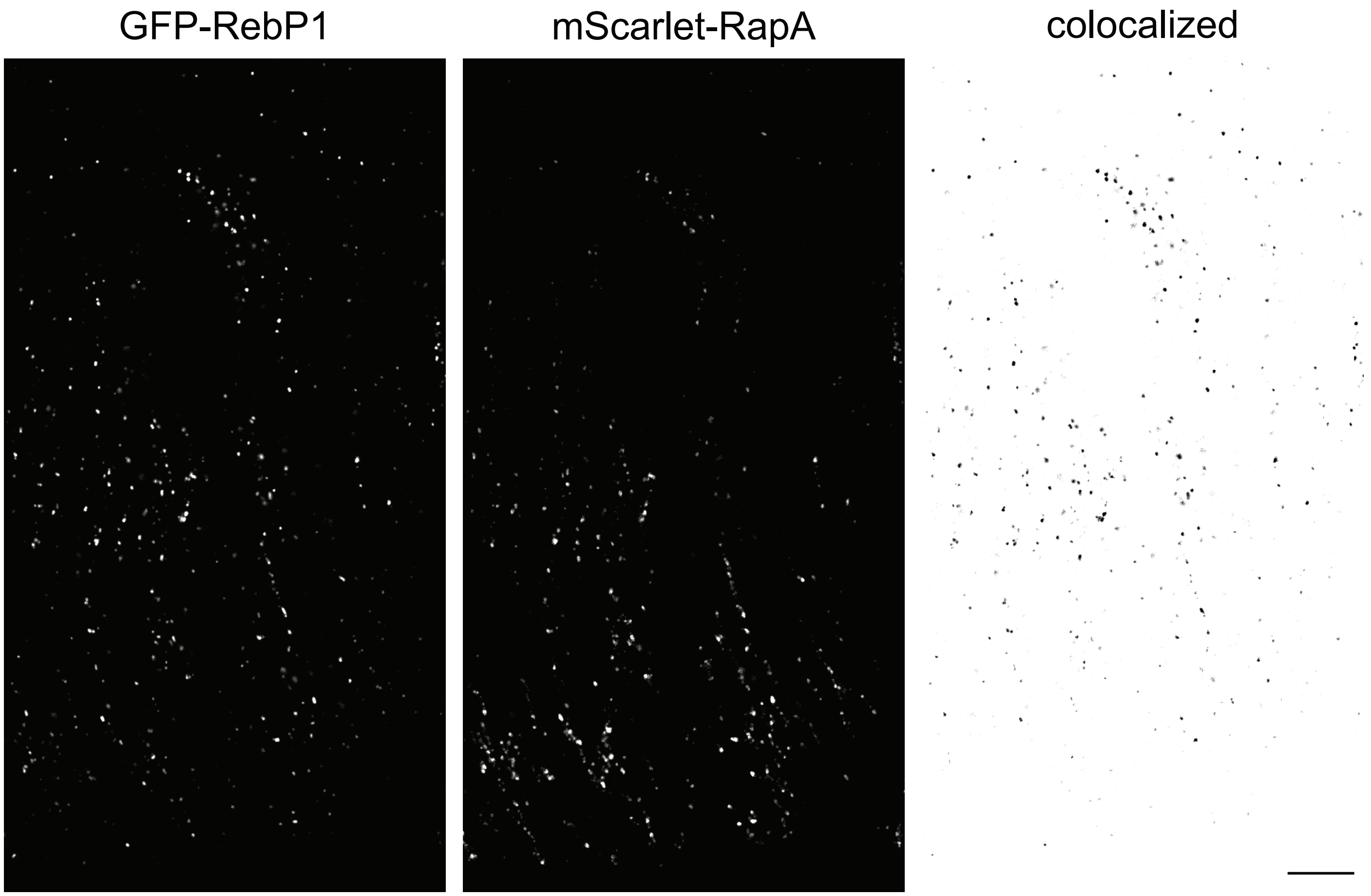

Supplementary Figure 5. Colocalization of GFP-RebP1 and mScarlet-RapA. Representative confocal image of a thin section prepared from three-day-old biofilms of the dual-labeled strain shown in **Figure 4b**. To visualize the degree of colocalization, we generated a binary mask of GFP-RebP1 in ImageJ. The mask was then applied (ImageJ image calculator, Subtract) to the mScarlet-RapA image leaving only the colocalized fraction of pixels as shown on the right. Images are representative of five fields of view captured from two experiments. Scale bar is 10 µm.

Supplementary Table 1: Strains

Strain	Number	Description	Source	Appears in figure
Pseudomonas aeruginosa				
UCBPP-PA14		Clinical isolate UCBPP-PA14.	(2)	2b-e, 3e-f, 4c-d, Table S2
PA14 Δ*pel*	LD82	PA14 with deletion in *PA14_24490 (pelB)-24560 (pelG).*	(3)	2b-e, S3, Table S2
PA14 Δpel ΔrebP1P2	LD2720	PA14 with deletion in *PA14_24490-24560* and *PA14_27640 (rebP1)-27640 (rebP2).* Made by mating pLD2934 into LD2720.	This study	S3
PA14 Δpel ΔrebP1P2:: *rebP1P2*	LD3026	PA14 Δ*pelB-G* Δ*PA14_27640 (rebP1)-27630 (rebP2)* with wild-type *PA14_27630-27640* complemented back into the site of deletion. Made by mating pLD3016 into LD2936.	This study	S3
PA14 P_{rebP1}-*mScarlet*	LD3224	PA14 with P_{rebP1}-*mScarlet* inserted at the *attB* site using pLD3210.	This study	3a, 4a-b
PA14 Δ*rcgAfecI2*	LD3137	PA14 with deletion in *PA14_27690 (fecI2) -27700 (rcgA)*. Made by mating pLD3136 into UCBPP-PA14.	This study	-
PA14 Δ*fecI2*	LD3144	PA14 with deletion in *PA14_27690 (fecI2)*. Made by mating pLD3142 into UCBPP-PA14.	This study	-
PA14 Δ*rcgA*	LD3145	PA14 with deletion in *PA14_27700 (rcgA)*. Made by mating pLD3143 into UCBPP-PA14.	This study	-
PA14 Δ*rcgAfecI2*:: *rcgA-fecI2*	LD3180	PA14 Δ*PA14_27700 (rcgA)-27690 (fecI2)* strain with wild-type *PA14_27700-27690* complemented back into the site of deletion. Made by mating pLD3179 into LD3137.	This study	-
PA14 Δ*rcgAfecI2* P_{rebP1}-*mScarlet*	LD3782	PA14 Δ*PA14_27690 (rcgA)-27700 (fecI2)* with P_{rebP1}-*mScarlet* inserted at the *attB* site using pLD3210.	This study	3a
PA14 Δ*fecI2* P_{rebP1}-*mScarlet*	LD3780	PA14 Δ*PA14_27690 (fecI2)* with P_{rebP1}-*mScarlet* inserted at the *attB* site using pLD3210.	This study	3a
PA14 Δ*rcgA* P_{rebP1}-*mScarlet*	LD3781	PA14 Δ*PA14_27700 (rcgA)* with P_{rebP1}-*mScarlet* inserted at the *attB* site using pLD3210.	This study	3a

PA14 Δ*rcgAfecI2*:: *rcgAfecI2* P_{rebP1}-*mScarlet*	LD3783	PA14 Δ*PA14_27700 (rcgA)-27690 (fecI2)* strain with wild-type *PA14_27700-27690* complemented back into the site of deletion. The strain also has P_{rebP1}-*mScarlet* inserted at the *attB* site using pLD3210.	This study	3a
PA14 MCS-*mScarlet*	LD3294	PA14 without a promoter driving *mScarlet* expression inserted at the *attB* site using pLD3208.	This study	3a, 4a-b
PA14 P_{rebP1}-*mScarlet* $P_{PA1/04/03}$-*gfp*	LD3657	PA14 with P_{rebP1}-*mScarlet* inserted at the *attB* site and $P_{PA1/04/03}$-*gfp* inserted at the *glmS* site by mating pLD3655 into LD3224.	This study	3b-d
PA14 Δ*rcgAfecI2* P_{rebP1}-*mScarlet* $P_{PA1/04/03}$-*gfp*	LD3786	PA14 Δ*PA14_27700 (rcgA)-27690 (fecI2)* with P_{rebP1}-*mScarlet* inserted at the *attB* site using pLD3210 and $P_{PA1/04/03}$-*gfp* inserted at the *glmS* site by mating pLD3655 into LD3782.	This study	3c-d
PA14 Δ*fecI2* P_{rebP1}-*mScarlet* $P_{PA1/04/03}$-*gfp*	LD3784	PA14 Δ*PA14_27690 (fecI2)*with P_{rebP1}-*mScarlet* inserted at the *attB* site and $P_{PA1/04/03}$-*gfp* inserted at the *glmS* site by mating pLD3655 into LD3780.	This study	3c-d
PA14 Δ*rcgA* P_{rebP1}-*mScarlet* $P_{PA1/04/03}$-*gfp*	LD3785	PA14 Δ*PA14_27700 (rcgA)* with P_{rebP1}-*mScarlet* inserted at the *attB* site and $P_{PA1/04/03}$-*gfp* inserted at the *glmS* site by mating pLD3655 into LD3781.	This study	3c-d
PA14 Δ*rcgAfecI2*:: *rcgA-fecI2* P_{rebP1}-*mScarlet* $P_{PA1/04/03}$-*gfp*	LD3787	PA14 Δ*PA14_27700 (rcgA)-27690 (fecI2)* strain with wild-type *PA14_27700-27690* complemented back into the site of deletion. The strain also has P_{rebP1}-*mScarlet* inserted at the *attB* site and $P_{PA1/04/03}$-*gfp* inserted at the *glmS* site by mating pLD3655 into LD3783.	This study	3c-d
PA14 Δ*rebP1P2*	LD2935	PA14 with deletion in *PA14_27640 (rebP1)-27630 (rebP2)*. Made by mating pLD2934 into UCBPP-PA14.	This study	3e-f, 4c-f
PA14 $P_{PA1/04/03}$-*mScarlet*	LD3765	PA14 constitutively expressing mScarlet. Made by mating pLD3433 into UCBPP-PA14.	This study	3f
PA14 Δ*rebP1P2* $P_{PA1/04/03}$-*mScarlet*	LD3766	PA14 Δ*PA14_27640 (rebP2)-27630 (rebP2)* constitutively expressing mScarlet. Made by mating pLD3433 into LD2935.	This study	3f
PA14 $P_{PA1/04/03}$-*mScarlet*	LD3295	PA14 with *lac*-derived constitutive *PA1/04/03* promoter driving mScarlet expression inserted at the *attB* site using pLD3293.	This study	4a-b

PA14 Δ*rebP1P2*::*rebP1P2*	LD3025	PA14 Δ*PA14_27640 (rebP1)-27630 (rebP2)* with wild-type *PA14_27640-27630* complemented back into the site of deletion. Made by mating pLD3016 into LD2935.	This study	4c-d
PA14 *gacA*::*Tn*	LD1560	*MAR2xT7* transposon insertion into *PA14_30650 (gacA)*.	(4)	4c-d
Arabidopsis thaliana				
Col-0			J. Reed, UNC-Chapel Hill	4a,c
Caenorhabditis elegans				
unc-44(e362)	LD3326		(5) M. Chalfie, Columbia University	4b,d
Escherichia coli				
UQ950	LD44	*E. coli* DH5α λ(*pir*) strain for cloning; F- Δ(*argF-lac*) 169φ80d*lacZ*58(ΔM15) *glnV44*(AS) *rfbD1 gyrA96*(NalR) *recA1 endA1 spoT thi-1 hsdR17 deoR λpir+*	D. Lies, Caltech	
BW29427	LD661	Donor strain for biparental conjugation; *thrB1004 pro thi rpsL hsdS lacZ* ΔM15RP4-1360 Δ(*araBAD*)*567* Δ*dapA1341*::[*erm pir*(wt)]	W. Metcalf, University of Illinois	
S17-1	LD2901	StrR, TpR, F-RP4-2-*Tc::Mu aphA::Tn7 recA λpir* lysogen	(6)	
ß2155	LD69	Helper strain. *thrB1004 pro thi strA hsdsS lacZ*ΔM15 (F′ *lacZ*ΔM15 *lacI*^{q} *traD36 proA*$^{+}$ *proB*$^{+}$) Δ*dapA*::*erm* (Erm^{r}) *pir*::RP4 [::kan (Km^{r}) from SM10]	(7)	
Saccharomyces cerevisiae				
InvSc1	LD676	*MATa/MATα leu2/leu2 trp1-289/trp1-289 ura3-52/ ura3-52 his3-Δ1/his3-Δ1*	Invitrogen	

Supplementary Table 2: Primers

Primer number	Sequence	Used for plasmid
LD2609	acgtacgtctcgagtctagatttaagaaggagatatacatatgagtaaaggagaagc	pLD3208
LD2635	actgactggagctcataaaacgaaaggcccagtctttcg	
LD2113	acgtacgtacACTAGTccagatcctgcagaacgtc	pLD3210
LD2114	acgtacgtacGAATTCgatgtgactccctgtgagtgaa	
LD1087	AGGGCCAATCGATAGAGTTT	
LD1088	TCTTCGTGATCTGAAGCCATT	
LD3139	acgtacgtacgcatgctg AGTAAAGGAGAAGAACTTTTCACTGG	pLD3655
LD3141	acgtacgtacgctagc GGCGGATTTGTCCTACTCAG	
LD2731	acgtacgtacACTAGTtatttagaaaaataaacaaataggggttccgc	pLD3293
LD2732	acgtacgtacGAATTCgcttaatttctcctctttaattctagatgtgtg	
LD2507	ggaattgtgagcggataacaatttcacacaggaaacagctCTACGATTGGGTGTCCTTGC	pLD3136 (LD2507-2510),<br><br>pLD1853 (LD2507 and LD2510)
LD2508	TGTTCACGCCACTACACCCGCGACGTTTCACAAGACAGA	
LD2509	TCTGTCTTGTGAAACGTCGCGGGTGTAGTGGCGTGAACA	
LD2510	aggcaaattctgttttatcagaccgcttctgcgttctgatAGCGCTTCGACGAACAAC	
LD2507	ggaattgtgagcggataacaatttcacacaggaaacagctCTACGATTGGGTGTCCTTGC	pLD3142
LD2521	TATGGATCGTCCGAATCAGCGCGACGTTTCACAAGACAGA	
LD2522	TCTGTCTTGTGAAACGTCGCGCTGATTCGGACGATCCATA	
LD2523	aggcaaattctgttttatcagaccgcttctgcgttctgatCTCGCTACCCTTTCCGAATA	
LD2525	ggaattgtgagcggataacaatttcacacaggaaacagctACATGTCTGGGCACTCCTG	pLD3143
LD2526	CACGCCACTACACCCTGCCTGTCACCCAGGTAACAGCC	
LD2527	GGCTGTTACCTGGGTGACAGGCAGGGTGTAGTGGCGTG	
LD2510	aggcaaattctgttttatcagaccgcttctgcgttctgat AGCGCTTCGACGAACAAC	
LD2191	ggaattgtgagcggataacaatttcacacaggaaacagctCGCGCGCAACTCTTCTAT	pLD2934 (LD2191-LD2194),<br><br>pLD3016 (LD2191 and LD2194)
LD2192	gagttttccgaccgcagtcCTGCTGACGGTGCTCAAAG	
LD2193	ctttgagcaccgtcagcagGACTGCGGTCGGAAAACTC	
LD2194	aggcaaattctgttttatcagaccgcttctgcgttctgatGAAATATCGGACAGCGATGC	

LD2507	ggaattgtgagcggataacaatttcacacaggaaacagct CTACGATTGGGTGTCCTTGC	pLD3136 (LD2507-LD2509), pLD3179 (LD2507 and 2509)
LD2508	TGTTCACGCCACTACACCCGCGACGTTTCACAAGACAGA	
LD2509	TCTGTCTTGTGAAACGTCGCGGGTGTAGTGGCGTGAACA	
LD2510	aggcaaattctgttttatcagaccgcttctgcgttctgat AGCGCTTCGACGAACAAC	
LD2811	acgtacgtGCTGAGC AGTAAAGGAGAAGCTGTGATTAAAG	pLD3433
LD2812	acgtacgtGCT GAGCAGTAAAGGAGAAGCTGTGATTAAAG	
LD2598	ggaattgtgagcggataacaatttcacacaggaaacagctGATTGCCGACCGCCTGGCCA	pLD3198
LD2599	GTTCTTCTCCTTTACTCAT ctagtaGTCCTGTGTGA ACGGCGGCGATCAGGGCA	
LD2600	GCTGCCCTGATCGCCGCCGTTCACACAGGACtactagATGAGTAAAGGAGAA GAACTTTT	
LD2601	ACTGCGGTTGGGAAGCTCATGCCGGATCCTTGGCCTGATTTGTATAGTTCA TCCATGCc	
LD2602	GGCATGGATGAACTATACAAATCAGGCCAAGGATCCGGCATGAGCTTCCC AACCGCAGT	
LD2603	caaattctgttttatcagaccgcttctgcgttctgatATGATGAGGGCGGTAATGAGGAG	
LD3350	cctgcaggtcgactctagag GCGACGTTTCACAAGACAGA	pLD3791
LD3356	acagcttctcctttactcatctagtaGTCCTGTGTGACCGGTGGTGGCTACGATT	
LD3357	AATCGTAGCCACCACCGGTCACACAGGACtactagatgagtaaaggagaagctgt	
LD3358	CTTGCCGAAGCCGAACATGCCGGATCCTTGGCCTGAtttgtatagttcatccatgcc	
LD3359	ggcatggatgaactatacaaaTCAGGCCAAGGATCCGGCATGTTCGGCTTCGGCAAG	
LD3355	cagctatgaccatgattacg ATGGATGTGCAGGACCATCT	
LD3221	ggaattgtgagcggataacaatttcacacaggaaacagctGTCCAGGTAGTGGCGAAACA	pLD3685
LD3222	GCTTCGGCAAGAAATCCGGCAAGGACACCCAATCGTAG	
LD3223	CTACGATTGGGTGTCCTTGCCGGATTTCTTGCCGAAGC	
LD3224	aggcaaattctgttttatcagaccgcttctgcgttctgat CCGCCTCACAGACTGCTC	

Supplementary Table 3: Plasmids

Plasmids	Number	Description	Source
pMQ30	LD621	Yeast-based allelic-exchange vector; *sacB*+, CEN/ ARSH, URA3+, GmR .	(8)
pLD3208	LD3208	MCS-*mScarlet* GmR, TetR flanked by Flp recombinase target (FRT) sites to resolve out resistance cassettes. Cloned by swapping gfp sequence with mScarlet (XhoI + SacI)	This study
pFLP2	LD743	Site-specific excision vector with cI857-controlled FLP recombinase encoding sequence, sacB, ApR.	(9)
pLD3210 (P_{rebP1}-*mScarlet*)	LD3210	487 bp of *rebP1* promoter sequence inserted at the MCS (SpeI and EcoRI) of pLD3208	This study
pLD3655	LD3655	*gfp* coding region cloned into pAKN69 plasmid replacing the *yfp* coding region excised with SphI and NheI	This study
pLD3293 (*mScarlet*+)	LD3293	*lac*-derived constitutive promoter PA1/04/03 inserted at the MCS (SpeI and EcoRI) of pLD2722	This study
pLD3136	LD3136	Δ*PA14_27700 (rcgA)-27690 (fecI2)* flanking fragments introduced into pMQ30 by gap repair cloning in yeast strain InvSc1	This study
pLD3142	LD3142	Δ*PA14_27690 (fecI2)* flanking fragments introduced into pMQ30 by gap repair cloning in yeast strain InvSc1	This study
pLD3143	LD3143	Δ*PA14_27700 (rcgA)* flanking fragments introduced into pMQ30 by gap repair cloning in yeast strain InvSc1	This study
pLD3179	LD3179	Full genomic sequence of *PA14_27700 (rcgA)-27690 (fecI2)* PCR fragment introduced into pMQ30 by gap repair cloning in yeast strain InvSc1. Verified by illumina sequencing.	This study
pLD2934	LD2934	Δ*rebP1P2* flanking fragments introduced into pMQ30 by gap repair cloning in yeast strain InvSc1	This study
pLD3016	LD3016	Full genomic sequence of *rebP1P2* and native promoter PCR fragment introduced into pMQ30 by gap repair cloning in yeast strain InvSc1. Verified by illumina sequencing.	This study
pLD3433	LD3433	*mScarlet* coding region cloned into pAKN69 plasmid replacing the *yfp* coding region that was excised with BlpI and NheI.	This study

SUPPLEMENTARY REFERENCES

1. Raymann, K., Bobay, L.-M., Doak, T. G., Lynch, M. & Gribaldo, S. A genomic survey of Reb homologs suggests widespread occurrence of R-bodies in proteobacteria. *G3* **3**, 505–516 (2013).

2. Rahme, L. G. *et al.* Common virulence factors for bacterial pathogenicity in plants and animals. *Science* **268**, 1899–1902 (1995).

3. Madsen, J. S. *et al.* Facultative control of matrix production optimizes competitive fitness in *Pseudomonas aeruginosa* PA14 biofilm models. *Appl. Environ. Microbiol.* **81**, 8414–8426 (2015).

4. Liberati, N. T. *et al.* An ordered, nonredundant library of *Pseudomonas aeruginosa* strain PA14 transposon insertion mutants. *Proc. Natl. Acad. Sci. U. S. A.* **103**, 2833–2838 (2006).

5. Brenner, S. The genetics of *Caenorhabditis* elegans. *Genetics* **77**, 71–94 (1974).

6. Simon, R., Priefer, U. & Pühler, A. A Broad Host Range Mobilization System for In Vivo Genetic Engineering: Transposon Mutagenesis in Gram Negative Bacteria. *Biotechnology* **1**, 784–791 (1983).

7. Dehio, C. & Meyer, M. Maintenance of broad-host-range incompatibility group P and group Q plasmids and transposition of Tn5 in Bartonella henselae following conjugal plasmid transfer from *Escherichia coli*. *J. Bacteriol.* **179**, 538–540 (1997).

8. Shanks, R. M. Q., Caiazza, N. C., Hinsa, S. M., Toutain, C. M. & O’Toole, G. A. *Saccharomyces cerevisiae*-based molecular tool kit for manipulation of genes from gram-negative bacteria. *Appl. Environ. Microbiol.* **72**, 5027–5036 (2006).

9. Hoang, T. T., Karkhoff-Schweizer, R. R., Kutchma, A. J. & Schweizer, H. P. A broad-host-range Flp-FRT recombination system for site-specific excision of chromosomally-located DNA sequences: application for isolation of unmarked *Pseudomonas aeruginosa* mutants. *Gene* **212**, 77–86 (1998).
