# Supplementary material for: Pseudomonas aeruginosa PA14 produces R-bodies, extendable protein polymers with roles in host colonization and virulence
Source: Nat Commun. 2021 Jul 29;12:4613. doi: 10.1038/s41467-021-24796-0 (PMC8322103; doi:10.1038/s41467-021-24796-0)
Supplement: Supplementary file 3 — Description of Additional Supplementary Files [file 41467_2021_24796_MOESM3_ESM.pdf]

Description of Additional Supplementary Files

File Name: Supplementary Data 1
Description: *Pseudomonas aeruginosa* strains that contain homologues of *rebP1* (PA14_27640), *fecI2* (PA14_27690) and the *reb* gene cluster PA14_27630-PA14_27700. Strains were identified with a nucleotide NCBI BLAST against 312 complete genomes in *Pseudomonas aeruginosa* (taxid: 287). 189 strains contained the *reb* cluster with *rebP1* and *fecI2*; in one strain the cluster lacked *fecI2*. 122 strains lacked the *reb* cluster, *rebP1* and *fecI2*.

File Name: Supplementary Data 2
Description: List of all 126 proteins (represented by two or more peptides) found in the SDS-insoluble fraction by tandem mass spectrometry.
