# Supplementary material for: Pseudomonas aeruginosa PA14 produces R-bodies, extendable protein polymers with roles in host colonization and virulence
Source: Nat Commun. 2021 Jul 29;12:4613. doi: 10.1038/s41467-021-24796-0 (PMC8322103; doi:10.1038/s41467-021-24796-0)
Supplement: Supplementary file 6 — Reporting Summary [file 41467_2021_24796_MOESM6_ESM.pdf]

nature research

Corresponding author(s): Lars Dietrich

Last updated by author(s): Apr 29, 2021

Reporting Summary

Nature Research wishes to improve the reproducibility of the work that we publish. This form provides structure for consistency and transparency in reporting. For further information on Nature Research policies, see our Editorial Policies and the Editorial Policy Checklist.

Statistics

For all statistical analyses, confirm that the following items are present in the figure legend, table legend, main text, or Methods section.

n/a	Confirmed	
☐	☒	The exact sample size (*n*) for each experimental group/condition, given as a discrete number and unit of measurement
☐	☒	A statement on whether measurements were taken from distinct samples or whether the same sample was measured repeatedly
☐	☒	The statistical test(s) used AND whether they are one- or two-sided<br>*Only common tests should be described solely by name; describe more complex techniques in the Methods section.*
☒	☐	A description of all covariates tested
☒	☐	A description of any assumptions or corrections, such as tests of normality and adjustment for multiple comparisons
☐	☒	A full description of the statistical parameters including central tendency (e.g. means) or other basic estimates (e.g. regression coefficient) AND variation (e.g. standard deviation) or associated estimates of uncertainty (e.g. confidence intervals)
☐	☒	For null hypothesis testing, the test statistic (e.g. *F*, *t*, *r*) with confidence intervals, effect sizes, degrees of freedom and *P* value noted<br>*Give P values as exact values whenever suitable.*
☒	☐	For Bayesian analysis, information on the choice of priors and Markov chain Monte Carlo settings
☒	☐	For hierarchical and complex designs, identification of the appropriate level for tests and full reporting of outcomes
☐	☒	Estimates of effect sizes (e.g. Cohen's *d*, Pearson's *r*), indicating how they were calculated

Our web collection on statistics for biologists contains articles on many of the points above.

Software and code

Policy information about availability of computer code

Data collection	Confocal microscopy data were collected with Zeiss Zen 2 software. Mass spectrometry data was collected using Waters MassLynx™ 4.1 Software (for Synapt G2) and Thermo Xcalibur™ 3.1 Software (for Q Exactive HF Orbitrap).
Data analysis	Geneious Prime 2020.2.4 and iTOL v6 were used for phylogenetic analysis. Zeiss Zen 2 and ImageJ/Fiji 1.53c with MicrobeJ 5.13l and JACoP 2.1.1 plugins were used for image analysis. Mascot V 2.5 was used for mass spectrometry analysis.

For manuscripts utilizing custom algorithms or software that are central to the research but not yet described in published literature, software must be made available to editors and reviewers. We strongly encourage code deposition in a community repository (e.g. GitHub). See the Nature Research guidelines for submitting code & software for further information.

Data

Policy information about availability of data

All manuscripts must include a data availability statement. This statement should provide the following information, where applicable:

- Accession codes, unique identifiers, or web links for publicly available datasets
- A list of figures that have associated raw data
- A description of any restrictions on data availability

All raw MS data files are publicly available at the MassIVE data repository under MassIVE submission # MSV000087657 (https://massive.ucsd.edu/ProteoSAFe/dataset.jsp?accession=MSV000087657).
Plasmids and strains generated during and/or analyzed during the current study are available from the corresponding author on reasonable request. Source data are provided with this paper.

Field-specific reporting

Please select the one below that is the best fit for your research. If you are not sure, read the appropriate sections before making your selection.

- [x] Life sciences
- [ ] Behavioural & social sciences
- [ ] Ecological, evolutionary & environmental sciences

For a reference copy of the document with all sections, see nature.com/documents/nr-reporting-summary-flat.pdf

Life sciences study design

All studies must disclose on these points even when the disclosure is negative.

Sample size	Sample sizes were chosen based on previous experience and in accordance to the standards in the field and at least three independent replicates were analyzed.
Data exclusions	No data were excluded.
Replication	Replicates were used as indicated and all findings are reflected in the figures.
Randomization	Randomization was not required in this study.
Blinding	Blinding was not required in this study.

Reporting for specific materials, systems and methods

We require information from authors about some types of materials, experimental systems and methods used in many studies. Here, indicate whether each material, system or method listed is relevant to your study. If you are not sure if a list item applies to your research, read the appropriate section before selecting a response.

Materials & experimental systems

n/a	Involved in the study	
x		Antibodies
x		Eukaryotic cell lines
x		Palaeontology and archaeology
x		Animals and other organisms
x		Human research participants
x		Clinical data
x		Dual use research of concern

Methods

n/a	Involved in the study	
x		ChIP-seq
x		Flow cytometry
x		MRI-based neuroimaging
